# A comprehensive analysis of miRNA/isomiRs profile of hydrosalpinx patients with interventional ultrasound sclerotherapy

**DOI:** 10.1371/journal.pone.0268328

**Published:** 2022-08-15

**Authors:** Zhengyi Cao, Bo Xu, Yan Wu, Kang Luan, Xin Du

**Affiliations:** 1 Reproductive Medicine Center, Hefei, Anhui, P.R. China; 2 Division of Life Sciences and Medicine, Reproductive and Genetic Hospital, The First Affiliated Hospital of USTC, University of Science and Technology of China, Hefei, China; China Agricultural University, CHINA

## Abstract

Hydrosalpinx is a chronic inflammatory condition with high recurrence rate, and it is reported among female population having fallopian tubal factor infertility. Previously, we have reported that interventional ultrasound sclerotherapy improves endometrial receptivity and pregnancy rate with negligible adverse effects in patients suffering from hydrosalpinx. During present investigation, we have used next generation sequencing (NGS) to characterize the isomiR profiles from the endometrium of patients suffering from hydrosalpinx before and after interventional ultrasound sclerotherapy. Our results indicated that miRNA arm shift and switch remained unaffected when compared in patients before and after interventional ultrasound sclerotherapy. We observed that isomiRs with trimming at 3’ and isomiRs with canonical sequences were lower in post-treatment than in pre-treatment group. Gene ontology (GO) annotation and KEGG pathway analysis revealed that the expression of mature mir-30 was significantly lower in the pre-treatment as compared to post treatment group while the expression of mir-30 isomiR was 4.26-fold higher in pre-treatment when compared with the post-treatment group. These different expression patterns of mir-30 mature miRNA and mir-30 isomiRs in two groups are affecting the physiological function of the endometrium. Our results suggested that differential isomiR distribution in hydrosalpinx patients before and after treatment plays an important role in hydrosalpinx incidence and can help in designing novel strategy for the treatment of hydrosalpinx in female population.

## Introduction

Hydrosalpinx refers to a pathological condition in which one or both fallopian tubes can be filled with a substantial amount of fluid, making them dilated and dysfunctional resulting in female infertility [1]. It has been documented that the presence of hydrosalpinx may affect embryo implantation and can reduce the endometrial receptivity during in vitro fertilization, thereby reducing the pregnancy rate by 50% and increasing the spontaneous abortion rate [2–5]. At present, salpingectomy and salpingoplasty are the most common invasive surgical treatments for patients with hydrosalpinx [6].

The aspiration of hydrosalpinx guided by transvaginal ultrasound is cheap, convenient, and non-invasive procedure that is in practice [7]. Previously, we had reported that ultrasound aspiration and sclerotherapy with 98% ethanol can improve the endometrial receptivity, reduce hydrosalpinx recurrence rate and improves the chances of successful in vitro fertilization and this treatment has no adverse effects on ovarian reserve [8,9].

Mature miRNA (miRNA) has an average length of 22 nucleotides, and they are responsible for the regulation of several important biological processes in cells during health as well as under pathological conditions. miRNAs are known to silence the target gene and to regulate the post-transcriptional gene expression through mRNA decay and translational repression [10]. Recently, miRNA is reported to be associated with hydrosalpinx-induced endometrial dysfunction [11,12]. miRNA isoforms (isomiRs) are the variants of miRNA that often differ from their corresponding reference mature sequences in stability and in some cases, they have distinct functions even from their close sequence relatives [13,14]. Formation of isomiRs is associated with modifications in precursor miRNAs that is usually done either by nucleotide addition and trimming at their 5’ or 3’ ends or by nucleotide modification at seed/out-seed regions by exoribonuclease and nucleotidyl-transferase [15]. The next-generation sequence (NGS) has revealed that these changes in miRNA and isomiRs that are generated as a result can affect the selection of target genes [16]. Keeping in view, the above-mentioned facts, in present investigation, we have used NGS to characterize isomiR profiles from endometrium of the patients suffering from hydrosalpinx before and after they underwent interventional ultrasound sclerotherapy in order to add in existing information about hydrosalpinx and to provide some novel isomiR profile related data that may lead to the development of novel strategies for the treatment of this common pathological condition in female population.

## Materials & methods

### Subjects and sample collection

In present study, three females suffering from hydrosalpinx were enrolled from 901st hospital of PLA Joint Logistic Support Force in Hefei (China) following their written informed consent. The samples were collected on July 25, July 29, and October 9 in 2019.The inclusion criteria were as follows: (1) all women were aged ws: years; (2) all women were undergoing their first attempt of IVF; (3) all women had normal ovulation and menstrual cycles; (4) baseline follicle-stimulating hormone < 12 mIU/L;(5) BMI < 28 Kg/m^2^. The exclusion criteria were severe endometriosis or fibroids, uterine anomaly, previous pelvic surgery, polycystic ovarian syndrome, smoking, alcohol or/and drug abuse. Hydrosalpinx was diagnosed via hysterosalpingography. All subjects received ultrasound sclerotherapy with 98% ethanol treatment. Ultrasound sclerotherapy was performed during the endometrial implantation window and endometrial scratching was performed for samples collecting.Endometrial samples were collected before and after treatment from each individual.

All the experimental procedures and protocols were approved by the Ethics Committees of the 901st hospital of PLA Joint Logistic Support Force (IRB No.: 901YY-2019-04).

### RNA isolation, small RNA sequencing and q-pcr

Total RNA was isolated from the endometrium of the patients with Trizol (Invitrogen, USA) and this RNA was used for NGS for miRNA/isomiRs profile analysis. The extracted RNA from the endometrium of hydrosalpinx patients was equally pooled before treatment (mark as pre-treatment). Endometrium samples were collected in a similar fashion as mentioned above after ultrasound sclerotherapy with 98% ethanol and equally pooled (mark as post-treatment). The pooled samples were dispersed by 15% denaturing polyacrylamide gel electrophoresis. According to Illumina’s protocol, miRNA libraries were constructed, and miRNA sequencing was performed on the Illumina Hiseq 2000(Illumina. USA).

cDNA was synthesized using PrimeScript^®^RT Master Mix (Takara Bio, Japan) according to the manufacturer’s instructions. qPCR was performed in ABI 7500 real time PCR system (Applied Biosystems, USA) using SYBR^®^Premix Ex Taq^™^ II Kit (Takara Bio, Japan). Primers were designed to span the introns. The specificity of the primers was confirmed by melting curve and agarose electrophoresis. Expression of the BTG1 gene (5’ AGCGGATTGGACTGAGCAG, 5’ GGTGCTGTTTTGAGTGCTACC) was calculated using the 2^-ΔΔCt^ method normalized to actin(5’ TGTTACCAACTGGGACGACA, 5’ GGGGTGTTGAAGGTCTCAAA).

### Computational analysis of sequencing data

An online tool, DeAnnIso (https://mcg.ustc.edu.cn/bsc/deanniso/), was used to detect and annotate IsomiR from small RNA sequencing data [17]. Briefly, the numbers of all reads mapped to the reference sequence was calculated as total reads count. The number of unique reads mapped to the specific sequence was calculated as unique tags. RPM (reads per million) was calculated as (*N*_*ref*_ / *N*_*total*_*)* × 10^6.^ (*N*_*ref*_*−*the number of reads mapped to the miRNA reference, *N*_*all*_ -a total number of reads mapped in the sample). Data generated was normalized to use RPM. miRanda (www.miRNA.org) was used to predict the targeted genes of isomiRs. The detail protocol for target gene prediction was same as in Zhang et al. (2016).

## Results

3 patients with confirmed hydrosalpinx underwent interventional ultrasound sclerotherapy. All patients tolerated the procedure well without any infection or other clinical complications. At follow up, 2 weeks after the procedure, the hydrosalpinx fluid disappeared completely.

### Overview of RNA sequencing data

**Fig 1:**
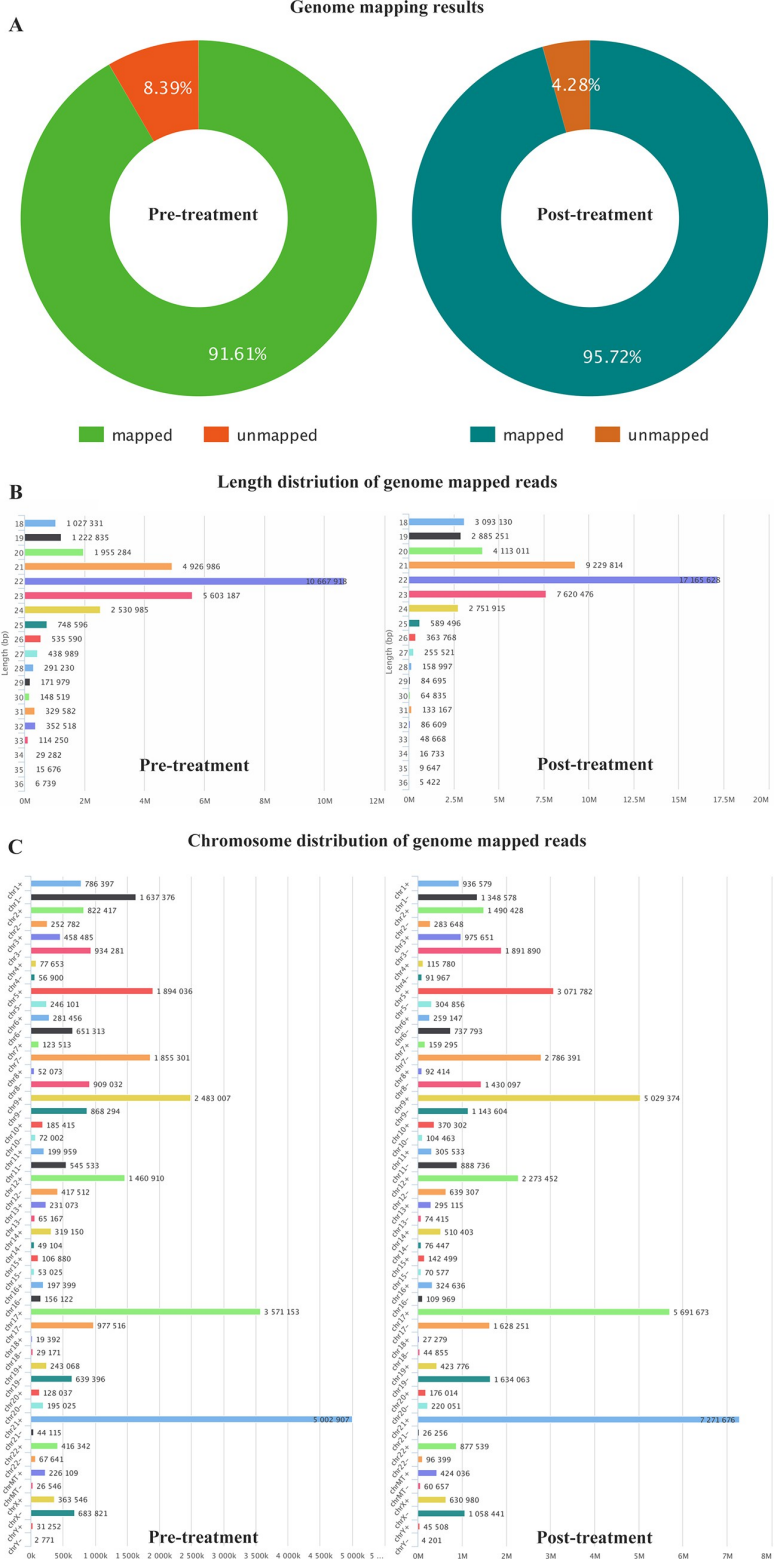
Overview of RNA sequencing data. (A) Genome mapping results; (B) Length distribution of genome mapped reads; (C) Chromosome distribution of genome mapped reads.

A total of 33,968,492 raw reads from the pre-treatment group and 50,854,626 from the post-treatment group were recorded following NGS. Total mapped reads were 1,727,423 and 2,304,190 were mapped unique tags, respectively (Fig 1A). The majority of total reads had average size of 22 nucleotides, while the two groups had size variation that ranged between 18 to 36 nucleotides. The length of most unique tags had size range of 18–24 nucleotides. Longest unique tag had 18 nucleotides in pretreatment while it was 22 nucleotides long in post treatment group (Fig 1B). The mapped reads ratio distributed in each chromosome were also analyzed. We observed that majority of the observed reads were in chromosome 21, followed by chromosome 17 and 9 respectively (Fig 1C).

### Analysis of known miRNA family expression

The known miRNA expression profile in pre-treatment and post-treatment groups was represented as total reads count of each miRNA family. The 20 most abundant miRNA families that accounted for almost 90% of the known miRNAs are listed in Table 1. It is worth mentioning that miRNA family miR-21 and let-7 represented almost 30% of the total reads that is consistent with the previously reported regulatory function of these miRNAs in endometrium [18,19].

**Table 1:** The Expression profile of 20 most highly expressed miRNAs in human endometrium.

*Statistics of miRNA family match results*					
*Pre-treatment*			*Post-treatment*		
Rank	Name	Reads Count	Rank	Name	Reads Count
1	mir-21	3069746	1	let-7	5962108
2	let-7	2922238	2	mir-21	5158743
3	mir-199	1990485	3	mir-10	3265375
4	mir-10	1755165	4	mir-27	2869560
5	mir-143	1704443	5	mir-143	2809974
6	mir-148	1680797	6	mir-199	2641711
7	mir-27	1393546	7	mir-148	2505838
8	mir-30	1245184	8	mir-30	1832314
9	mir-26	714914	9	mir-26	1480242
10	mir-8	354585	10	mir-8	532156
11	mir-25	298343	11	mir-25	419967
12	mir-23	178495	12	mir-23	325297
13	mir-17	145602	13	mir-126	272410
14	mir-103	120175	14	mir-3074	233482
15	mir-146	120136	15	mir-451	229795
16	mir-126	117587	16	mir-28	177968
17	mir-3074	112641	17	mir-146	174413
18	mir-451	109527	18	mir-17	164898
19	mir-101	108585	19	mir-34	150474
20	mir-34	105381	20	mir-127	145788

### Analysis of isomiRs identification and expression

**Fig 2:**
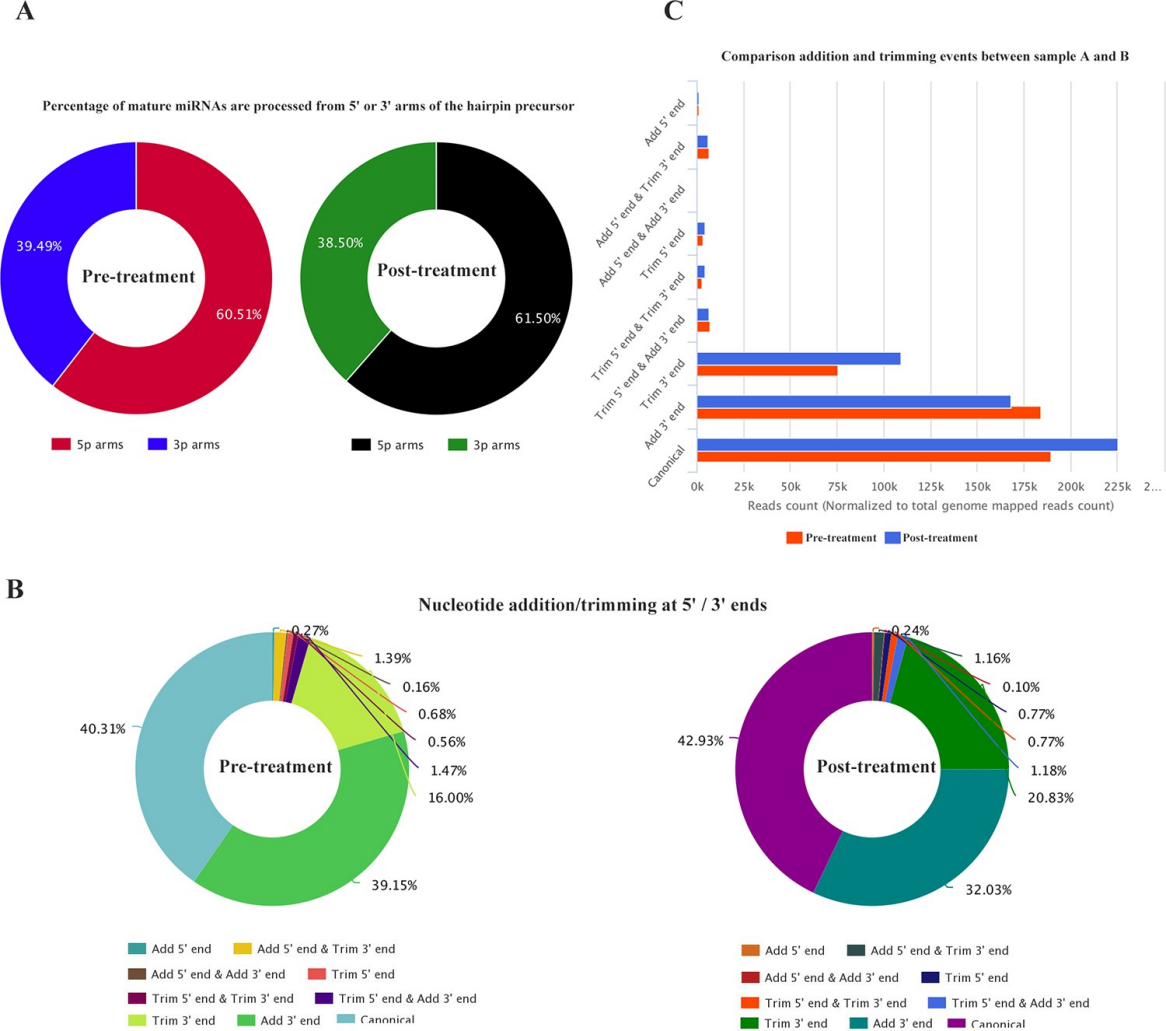
isomiR identification and expression analysis. (A) Percentage of mature miRNAs that are processed at 5’ or 3’ arms of the hairpin precursor; (B) Percentage of variants type with nucleotide addition/trimming at 5’ or 3’ ends; (C) Comparison of addition and trimming events between pre and post treatment groups.

We identified 2261 unique tags isomiRs in pre-treatment and 2635 unique tags isomiRs in post-treatment group. 60.51% of 5’ and 39.49% of 3’ of the miRNA precursors were found modified in pre-treatment group while 61.50% and 38.50% 5’ and 3’ of the miRNA precursors were modified in post-treatment group (Fig 2A). A similar percentage of arm processing was observed in two groups as we discussed for end modifications of miRNA indicating that there were no dramatic changes in miRNA arm shift and switch between the two groups.

Nucleotide addition/trimming at 5’ / 3’ ends was also analyzed and compared between the two groups (Fig 2B). Canonical sequences were most abundant, followed by isomiRs with addition at 3’end and trimming at 3’end (Fig 2C). We observed that isomiRs with trimming at 3’ and canonical sequences were less abundant in post-treatment than in pre-treatment group. For example, mir-223 and mir-506 isomiRs were lower in post-treatment group (S1 Table). Gene ontology (GO) annotation showed that the isomiRs with trimmed 3’ were not enriched in any biological process under investigation.

**Fig 3:**
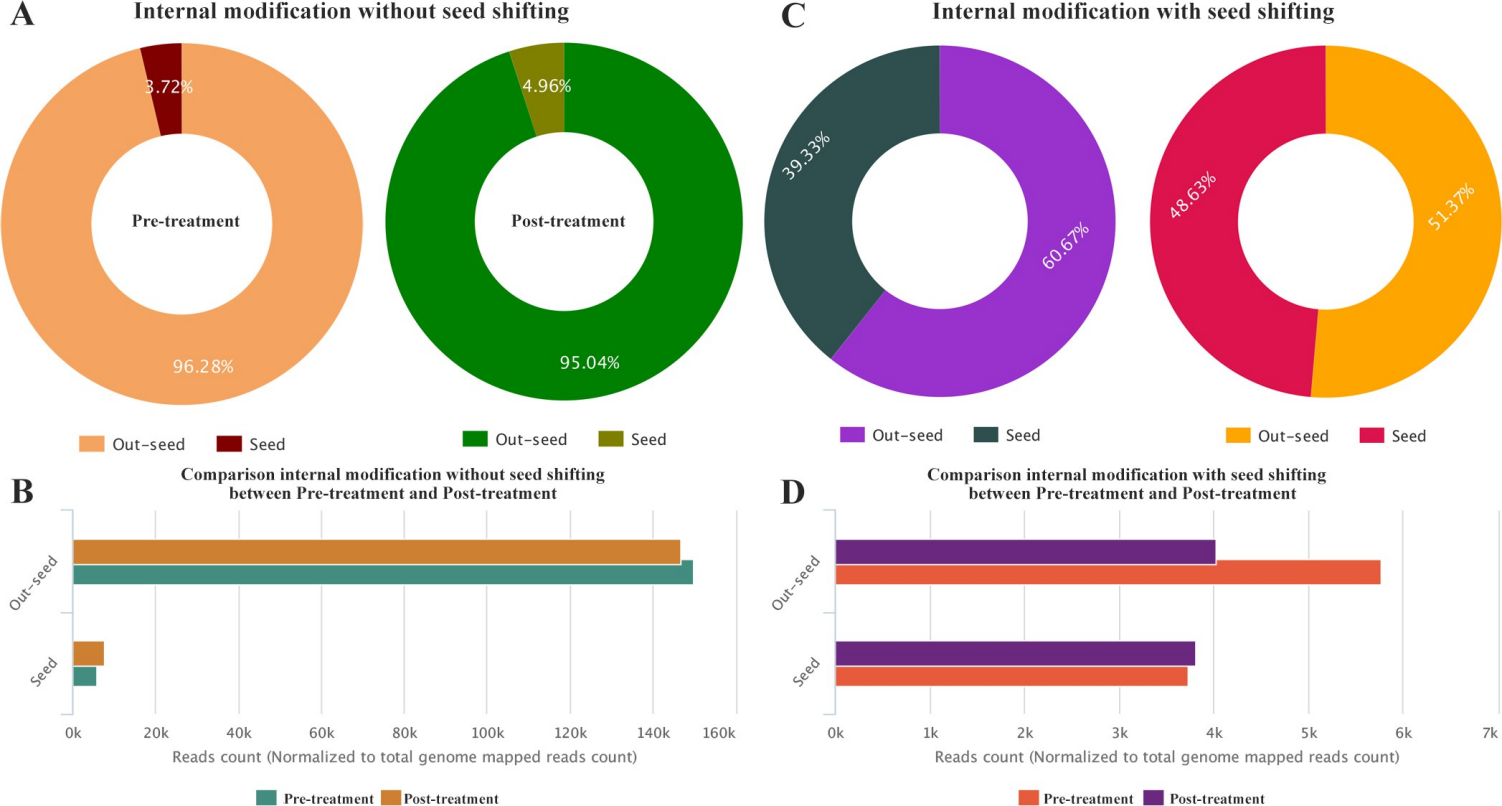
Distribution of internal modification in isomiRs. (A) Percentage of internal modification without seed shifting (B) Comparison of internal modification without seed shifting between pre-treatment and post treatment groups; (C) Percentage of internal modification with seed shifting; (D) Comparison internal modification with seed shifting between pre-treatment and post treatment groups.

Internal modification at the seed region or out-seed region in miRNA may shift the seed sequence. Seed sequence modification with variations or shifting has been shown to have functional consequences as they can change the target genes of miRNA. Therefore, we focused our analysis on internal modification with seed shifting. In pre-treatment group, we observed that 39.33% modification on seed region and 60.67% modifications on out-seed region contributed to the seed shifting. While in post-treatment group, 51.37% of seed shifting was caused by modification on the out-seed region (Fig 3). It was further observed that normalized to total genome mapped reads count and the modification on the out-seed region was much higher in pre-treatment (RPM = 5760) than in post-treatment group (RPM = 4020). Considering internal modification in the out-seed region, a trend of difference between the two groups was observed (Fig 3).

**Fig 4:**
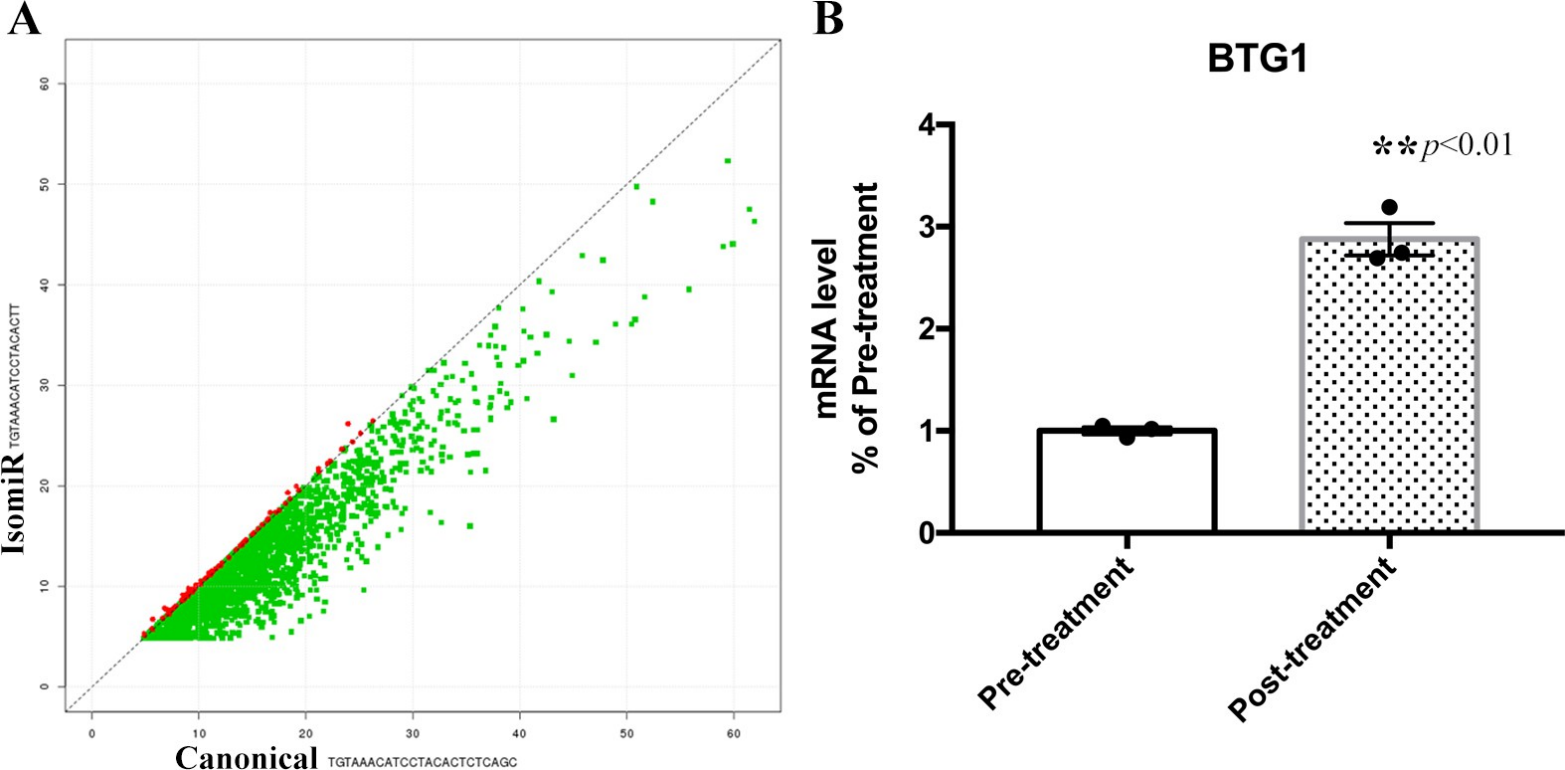
Scatter plot analysis showing target selection for mir-30 isomiRs. (A)Red color presenting the target of isomiR sequence: TGTAAACATCCT-ACACTT. Green pots show the target of canonical sequence: TGTAAACAT-CCTACACTCTCAGC. (B) BTG1 mRNA expression level was significantly increased in the post-treatment group (Values are mean ± SEM; pair t-test **p < 0.01).

We have also explored the expression of the isomiRs within the out-seed region modification and compared them between pre-treatment and post-treatment groups (S2 Table). Parameters were set to greater than 2-fold for expression and P value<0.05. The expression of mature mir-30 was significantly lower in the pre-treatment as compared to post treatment group (fold change = 0.4870, p < 0.001). However, the expression of mir-30 isomiR was 4.26-fold higher in pre-treatment when compared with post-treatment group (S2 Table). These different expression patterns of mir-30 mature miRNA and mir-30 isomiRs in two groups are affecting the physiological function of the endometrium. A scatter plot analysis was performed as well to report the different selection of Mir-30 isomiRs (Fig 4A). GO annotation and KEGG pathway analysis revealed that the miR-30 isomiRs is involved in regulating biological process (GO:0006552~leucine catabolic process;GO:0045475~locomotor rhythm;GO~0045603:positive regulation of endothelial cell differentiation) and molecular function (GO:0004177~aminopeptidase activity; GO:0004485~methylcrotonoyl-CoA carboxylase activity; GO:0017163~basal transcription repressor activity; GO:0070097~ delta-catenin binding) indicating that miR-30 isomiRs cluster plays a key role in endothelial cell differentiation by regulation of BTG1 (Table 2). We examined the expression of BTG1 mRNA level in endothelial tissue before and after treatment. Consistent with our predicted results, because of the negative regulation by mir-30 isomiRs, the expression of btg1 gene was significantly increased in the post-treatment group.

**Table 2:** Predicted targets of mir-30 isomiRs by gene ontology analysis.

*GO number*	*GO biological process*	*Target genes*	*Enrichment fold*	*P value*
*biological process*				
GO:0006552	leucine catabolic process	MCCC2	155.9222222	0.00654
GO:0045475	locomotor rhythm	NPAS2	103.9481481	0.00980
GO:0045603	positive regulation of endothelial cell differentiation	BTG1	311.8444444	0.00327
*molecular function*				
GO:0004177	aminopeptidase activity	DPP8 ENPEP	18.20848485	0.00572
GO:0004485	methylcrotonoyl-CoA carboxylase activity	MCCC2	136.5636364	0.00744
GO:0017163	basal transcription repressor activity	MDM2	136.5636364	0.00744
GO:0070097	delta-catenin binding	PTPRT	136.5636364	0.00744

## Discussion

Hydrosalpinx is a pathological condition that affects female fertility either by affecting the endometrium of the mother or by affecting the embryo. Hydrosalpinx is a fluid secreted by epithelial cells and blood plasma and it is supposed to reduce the endometrial receptivity by causing inflammation to it. Hydrosalpinx is also known to affect the process of egg-sperm union and embryo implantation during in vitro fertilization [4,20]. However, the molecular mechanism of hydrosalpinx is still unclear. Recently, it was reported that miRNA are plays important role in endometrial receptivity. miRNA is a type of endogenous small RNA that plays a variety of important roles in a cell as they target multiple genes and regulate their functions [21,22]. miRNA. is produced from a single-stranded RNA precursor with a hairpin structure of about 70–90 bases in size after being processed by Dicer enzyme [23,24]. Recently, NGS has revealed isomiRs, that refer to be the variants of mature miRNA, can arise from the same RNA precursor. The isomiRs can regulate different target genes than miRNAs that increased the diversity and complexity of the reactions regulated by miRNA [14].

In present study, next-generation sequencing technology was used to screen for the differential expression of isomiRs in the human endometrial sample. Our results suggested that the isomiRs expression profiles varied significantly in patients suffering from hydrosalpinx following sclerotherapy with 98% ethanol treatment. IsomiRs sequences were different from the mature miRNA sequences at 5’or 3’ addition/trimming or seed sequence region. It is an established fact that preferential expression of 3′ or 5′ of mature miRNA arm are highly dynamic process that affect its action [25]. Our results showed that the percentage of miRNA precursors that were processed from their 5’ or 3’ arm was almost same in both pre- and post-treatment groups (Fig 2). isomiRs trimmed at 3’end and with canonical sequences were lower in post-treatment group. Go annotation analysis revealed that the trimmed 3’ isomiRs were not enriched on differential target selection and on consequent biological process. These observations are indicating that the isomiRs with seed-shifting should be considered in cases where normal endometrium functions are compromised. Modification in the seed region implied changes in regulatory target genes., when the change in the out-seed region represents the change in regulatory strength. In this study, comparison of internal modification with seed shifting between pre-treatment and post-treatment revealed that the out-seed region was modified more in pre-treatment group. These modifications that occur in the outseed region lead to seed shifting. This implies that miRNA-regulated target genes also changed. Then these target gene alterations may be the reason for the occurrence of hydrosalpinx and the impact of IVF clinical outcome.

We found that expression of mir-30 isomiRs was significantly different when compared between pre- and post-treatment groups and this isomiR is associated with endothelial cell differentiation. We found that the expression of mature mir-30 was lower in the pre-treatment group, however the isomiRs mir-30 were highly enriched in the pre-treatment. It has been previously reported that miRNA-30 deficiency can affect endometrial receptivity and endometrium function. [26–28]. So the reduced expression of mature mir-30 in pre-treatment group may be the reason for the bad clinical outcome of IVF due to poor endometrial receptivity in hydrosalpinx patient.

the isomiRs mir-30 were highly enriched in the pre-treatment group. We identified the genes and biological process that could potentially be targeted by mir-30 isomirs using GO annotation and KEGG pathway analysis. GO annotation for the predicted genes of miRNAs pointed mir-30 isomiRs may affect GO:0045603: positive regulation of endothelial cell differentiation by regulation of Btg1 (p value = 0.0032).As known, the endothelial cells was contributed to the development and physiological function of the endometrium [29,30]. To verify this prediction, we examined the mRNA expression of BTG1 gene in endothelial tissues before and after treatment. The mRNA level of BTG1 gene was decreased in the pre-treatment group, which demonstrated the negative regulatory effect of mir-30 isomirs on BTG1 gene expression. The BTG1 gene is closely related to endothelial cell differentiation. This observation can be a partial explanation that how sclerotherapy is effective in treatment of hydrosalpinx. After sclerotherapy, mir-30 isomirs expression was reduced, resulting in elevated BTG1 expression, which promoted endothelial cell differentiation and repaired endothelial cell damage caused by hydrosalpinx. There are already some reports regarding the role of isomiRs in hydrosalpinx. It has been reported that miR-133b promotes *HOXA10* gene expression via target SGK1 to attenuate the hydrosalpinx-induced impairment of embryo attachment in vitro [31].

## Conclusions

In conclusion, for the first time, we are reporting endometrium miRNA/isomiRs expression profiles of hydrosalpinx patients before and after interventional ultrasound sclerotherapy. mature mir-30 played a critical role in regulating the receptivity of the endometrium. In contrast, mir-30 isomiRs exerts a regulatory effect by affecting more new target genes The GO annotation and KEGG pathway analysis for the predicted miRNA targets demonstrate that mir-30 isomiRs is involved in endothelial cell differentiation by BTG1. This data will facilitate the understanding of the mechanism of hydrosalpinx and in explaining that how sclerotherapy applies its positive effects in IVF outcome, and this data will help in developing novel strategies for the treatment of patients suffering hydrosalpinx.

## Supporting information

S1 Table Expression of the isomiRs with Trim 3’ modification between pre-treatment and post-treatment. (XLSX)

S2 Table Expression of the isomiRs with the out-seed region modification between pre-treatment and post-treatment. (XLSX)
